# The effects of emotional states and traits on time perception

**DOI:** 10.1186/s40708-018-0087-9

**Published:** 2018-08-20

**Authors:** Katie A. Lehockey, Andrea R. Winters, Alexandra J. Nicoletta, Taylor E. Zurlinden, Daniel E. Everhart

**Affiliations:** 1 MedStar National Rehabilitation Hospital, Washington, DC USA; 2 Department of Psychology, Rawl Building, East Carolina University, Greenville, NC 27858 USA

**Keywords:** Time perception, Emotion, Personality traits, BIS/BAS

## Abstract

**Background:**

Models of time perception share an element of scalar expectancy theory known as the internal clock, containing specific mechanisms by which the brain is able to experience time passing and function effectively. A debate exists about whether to treat factors that influence these internal clock mechanisms (e.g., emotion, personality, executive functions, and related neurophysiological components) as arousal- or attentional-based factors.

**Purpose:**

This study investigated behavioral and neurophysiological responses to an affective time perception Go/NoGo task, taking into account the behavioral inhibition (BIS) and behavioral activation systems (BASs), which are components of reinforcement sensitivity theory.

**Methods:**

After completion of self-report inventories assessing personality traits, electroencephalogram (EEG/ERP) and behavioral recordings of 32 women and 13 men recruited from introductory psychology classes were completed during an affective time perception Go/NoGo task. This task required participants to respond (Go) and inhibit (NoGo) to positive and negative affective visual stimuli of various durations in comparison to a standard duration.

**Results:**

Higher BAS scores (especially BAS Drive) were associated with overestimation bias scores for positive stimuli, while BIS scores were not correlated with overestimation bias scores. Furthermore, higher BIS Total scores were associated with higher N2d amplitudes during positive stimulus presentation for 280 ms, while higher BAS Total scores were associated with higher N2d amplitudes during negative stimuli presentation for 910 ms.

**Discussion:**

Findings are discussed in terms of arousal-based models of time perception, and suggestions for future research are considered.

## Introduction

### Time perception theory

#### Time perception theory history

Scalar expectancy theory utilizes a temporal information processing model, which suggests that an internal biological clock underlies a person’s ability to perceive time. This clock creates neuronal pulses, which are regulated by a theorized pacemaker. When attention is focused on the passage of time, a “switch” is flipped on and the number of accumulated pulses is counted until a signal is raised when some target interval duration is reached; this number is simultaneously held in reference memory. Subsequent judgments on the passage of time are made by comparing (comparator) the number of pulses being held in working memory with the value stored in reference memory [1–3].

Previous studies pertaining to how each of the aforementioned devices (i.e., the internal clock, the working-memory store, the reference memory store, and the comparator) works suggest that the use of external stimuli or internally activating factors may alter performance on time perception tasks. For example, it is thought that the pacemaker’s rate can be altered by factors such as body temperature [4] and pharmacological drugs [5] and by manipulating arousal. Treisman et al. [6] proposed a model that supports a relationship between increased arousal levels and underestimations of time.

Other models incorporate the concept of “attention” as an important component of time perception. For example, Zakay and Block [7] added the concept of a “gate” that lies between the pacemaker and the switch that mediates the effects of attention. As more attentional resources are allocated to tracking time, the gate opens wider, allowing more pulses to pass through to the accumulator [7]. Findings from this research suggest that time estimation is influenced by the amount of cognitive demand. Specifically, more demanding tasks are associated with shorter time duration estimations.

### Time perception, emotion, and personality traits

It is clear that time perception is affected by both arousal and attention and that emotion influences both of these variables [8, 9]. From an arousal perspective, emotional stimuli may lead to overestimations in time perception via a faster pacemaker rate. Attentional models, however, suggest that emotional stimuli may distract from temporal information processing, thereby reducing the amount of temporal pulses emitted, resulting in underestimations in time perception.

Past research has indicated that perceived durations of emotionally arousing events are usually distorted according to valence when compared to neutral events [10–14]. Generally, as arousal increases with the presentation of emotional stimuli, time estimations also increase. Negative valence, but not positive valence, is also generally correlated with overestimations.

The influence of emotional state on the perception of time has been studied among different normal populations. Notably, evidence of a double mechanism comprised of an approach–withdrawal attentional element and an appetitive–aversive emotional element has been supported, and its interaction with two primary components of emotion (affective valence and level of arousal) seems to play a role in evaluation of perceived time [10]. For example, people tend to overestimate negative compared to positive emotional stimuli if stimuli are highly arousing, while people tend to judge negative emotional low-arousal stimuli as shorter compared to positive low-arousal emotional stimuli during verbal estimation and temporal reproduction tasks. However, in this study, no overestimations were observed compared to real time, which Angrilli et al. [10] explained as a function of the complexity of the task used.

Personality traits, and in particular those that are associated with approach- and withdrawal-related behavior, may also have a relationship with time perception, though to date there is little research within this area. One such way to study personality traits, as they pertain to time perception, is through the use of the behavioral inhibition system/behavioral activation system (BIS/BAS), which is the major focus of this study. These systems are thought to have distinct neural pathways and are typically examined via self-report scales [15]. The BAS is associated with positive affect and approach behavior. It is also associated with at least one negative emotion, anger, due to its influence on approach motivation tendencies [16]. Neurophysiologically, BAS is linked to the mesolimbic dopaminergic pathway [17]. The BIS, on the other hand, is associated with negative affect and withdrawal behavior. BIS seems to be modulated by adrenergic and serotonergic pathways [17]. BIS and BAS strength is associated with right and left frontal lobe activation, respectively [18]. These findings are generally in line with the valence hypothesis of emotion, which posits that the brain processes emotion in an asymmetric manner according to valence, with the left hemisphere specializing in the experience of positive emotionality and the right hemisphere specializing in negative emotion [19]. Some inconsistent baseline asymmetry findings from studies using anger as an emotional factor, which is considered to be negative in valence, led to the consideration of the approach–withdrawal model of emotion. The approach–withdrawal model posits that the left and right frontal lobes are specialized for processing emotions that involve approach and withdrawal behaviors, respectively [16, 20].

Others have offered various theories concerning personality traits and the resultant effects on behavior. Gray’s [21] reinforcement sensitivity theory is comprised of three fundamental emotion systems: the behavioral activation system, the fight-flight-or-freeze system, and the behavioral inhibition system. Each system is associated with neural activity and neurotransmitters, including dopamine, which is of particular interest in time perception research as it plays an important role in motor movement timing.

Dopamine is also associated with feelings of pleasure and is used by the brain to reinforce behaviors associated with seeking out certain pleasurable experiences. Dopamine is thought to play a central role in the motivation system called BAS, which is sensitive to indications of reward, nonpunishment, and escape from punishment, causing a person to engage in goal-oriented behavior [15]. According to Gray’s reinforcement sensitivity theory, BAS is also thought to be responsible for the experience of positive emotions [15, 22]. In an electrophysiological study using positive, negative, and neutral emotional stimuli, people who rated high on the BAS scale had a significant and more intense response to positive emotional stimuli than to negative or neutral stimuli [22]. It has been found that people who have high BAS scores have increased left frontal activation [23], especially when presented with positive emotional stimuli [22].

Another component of Gray’s theory is the BIS, which is associated with anxiety, and is sensitive to signals of punishment, nonreward, and novelty [15]. It has been found that people who score high on BIS have greater right frontal activation in EEG studies [17, 22, 24]. People who score high on BIS are thought to experience more negative affect than those people who score low on BIS.

### Electrophysiology, time perception, and inhibition

One way to gain insight into any cognitive or emotional event that occurs at the subsecond level is to examine event-related potentials, or ERPs. ERPs are voltage changes that occur as a result of the brain’s response to a presented stimulus, and are thought to represent post-synaptic changes in neurons [25]. ERPs are recorded from a participant via electrodes evenly distributed across the scalp, while the participant engages in an experimental task. Positive and negative deflections of voltage (e.g., N1, P1, N2, P2) are of particular interest in cognitive neuroscience research, as are the latencies in milliseconds and amplitudes in microvolts of these deflections.

An aspect of executive function that is important in timing in conversations and withholding inappropriate responses is inhibition. Inhibition has been studied electrophysiologically using a Go/NoGo ERP task. In this type of task, participants are presented with target and nontarget stimuli and are asked to refrain from responding after the presentation of nontarget stimuli. Two ERP components are usually of interest in this kind of study, namely the N2 and P3 [26, 27].

The N2 is a frontal negative displacement that usually occurs between 200 and 300 ms after stimulus presentation. The P3 is a fronto-central positive displacement that usually occurs between 300 and 500 ms after stimulus presentation. The N2 component is thought to reflect inhibition on a premotor level [28], while the P3 component is thought to reflect motor inhibition, or the evaluation of inhibitory processes [26, 29]. A right preponderance of activity has been recorded on occasion for both the N2 and P3 [27]. Orbitofrontal and inferior anterior cingulate cortices (ACC) are thought to mediate the generation of these ERP components [26, 30].

### Purpose and hypotheses

To date, the relationships between time perception, emotion, and personality traits have not been systematically examined. The present study utilized a Go/NoGo time perception task using emotional stimuli to test the effect of emotional valence on time perception. Self-reported personality characteristics using the BIS/BAS scales and inhibitory neural correlates derived from ERPs were also examined. The purpose of the present study was to:

1. Examine the relationship among levels of BIS/BAS, affect, and perceived stimulus duration using behavioral and self-report measures. Since visual emotional stimuli elicit higher arousal levels, it was hypothesized that participants would overestimate durations of emotional stimuli compared to neutral stimuli. More specifically, higher self-reported BAS scores would be associated with the tendency to overestimate the amount of time that positive stimuli were presented since previous findings indicated higher BAS scorers had more intense responses to positive stimuli [22]. Furthermore, self-reported BIS scores would be associated with the tendency to overestimate the amount of time that negative stimuli were presented.
2. Use the Go/NoGo paradigm to compare the associations between the variables of BIS/BAS, stimulus duration, stimulus valence, and the inhibitory N2 ERP component. It was hypothesized that N2 amplitudes during the presentation of NoGo stimuli would be larger than those observed during Go stimuli. The N2 component was also expected to be different for participants who scored higher on BAS compared to participants who scored higher on BIS. With regard to stimulus valence, higher scores on BAS would be associated with larger N2 amplitudes for positive NoGo stimuli, while higher scores on BIS would be associated with larger N2 amplitudes for negative NoGo stimuli.

## Methods

### Participants

Based on a priori power analysis to detect large effects with 80% power using GPower 3.1, 45 right-handed volunteers aged 18 years and older (*M* = 19.78, SD = 4.1) from East Carolina University were recruited using the undergraduate psychology participant pool. Of these participants, 32 were women and 13 were men. All participants had normal or corrected-to-normal vision and no prior significant neurological or psychiatric history. Participants received extra credit in a psychology course for participation.

### Questionnaires

Participants completed several self-report measures before the experimental procedure. Carver and White’s [15] BIS/BAS scales were completed by the participants as a way to measure behavioral inhibition and behavioral activation of each participant, and the Lateral Preference Inventory was administered to assess for handedness and other features of lateral preference (i.e., eye, ear, leg) [31]. The behavioral inhibition scale (BIS) and behavioral activation scale (BAS) are comprised of 20 items which span four domains: BIS, BAS reward responsiveness, BAS Drive, and BAS fun seeking. The BIS scale is made up of seven items that measure sensitivity to withdrawal behavior and expectations of punishment. The BAS scales are made up of 13 items which measure anticipation of reward, motivation toward desired goals, and desire to approach novel situations with expectation of reward. Participants respond to each item using a 4-point Likert scale (1 indicating “strongly agree” and 4 indicating “strongly disagree”). The BIS/BAS scales possess decent internal consistency with alpha coefficients ranging from .66 to .76, and comparable test–retest reliability with test–retest coefficients ranging from .68 to .72.

Other self-report measures that were administered include the Barratt Impulsiveness Scale, the Mini-IPIP Scales, and the Sensation-Seeking Scale. These additional measures were included for exploratory purposes, in order to understand how impulsivity, core personality characteristics, and the propensity toward sensation seeking, respectively, may affect time perception.

The Barratt Impulsiveness Scale is a reliable measure of impulsivity with three factors (nonplanning, motor impulsivity, and attention impulsivity) in both normal and clinical populations [32]. The 30-item self-report instrument was originally developed as part of a larger attempt to relate anxiety and impulsiveness to psychomotor efficiency. It contains questions about everyday behavior such as whether individuals make comments “without thinking” and whether they switch jobs frequently or feel “restless in lectures.”

The Mini-IPIP is a short form of the 50-item international personality item pool-five-factor model measure that is used to survey the big five personality traits; it has demonstrated consistent convergent, discriminant, and criterion-related validity [33]. For this self-administered measure, respondents are instructed to read 20 phrases describing people’s behavior. Next, respondents rate themselves using 7-point Likert scale with varying degrees of agreement ranging from “1”—*Disagree Strongly*, to “7”—*Agree Strongly*. Consisting of four questions per factor, the scale was developed for circumstances in which lengthier personality measures may not be feasible. Nevertheless, the Mini-IPIP has been shown to be a valid and reliable measure of the big five factors of personality (neuroticism, extraversion, intellect/imagination, agreeableness, and conscientiousness) with notable internal consistency alphas at or > .60.

The Sensation-Seeking Scale is a 40-item questionnaire that is comprised of four different subscales: Thrill and Adventure Seeking (TAS), Disinhibition (Dis), Experience Seeking (ES), and Boredom Susceptibility (BS). The Sensation-Seeking Scale has demonstrated satisfactory internal reliability when total scores are considered, but when the subscales (Thrill and Adventure Seeking, Experience Seeking, Disinhibition, and Boredom Susceptibility) are considered separately, some concern is raised with regard to each of their reliabilities, especially considering its use of dated language and examples of sensation-seeking activities [34].

### Equipment and stimuli

The control and presentation of the experimental stimuli and recording of participants’ responses were managed with SCAN 4.5 software (Compumedics Neuroscan, El Paso, TX). The stimuli that were presented to represent duration conditions consisted of three types of pictures (positive, negative, or neutral) selected from the International Affective Pictures System (IAPS), which were matched for valence and arousal [35]. All items were matched for luminance and size. The pictures selected for this study were inanimate art and household objects. Event-related potentials were recorded during stimuli presentation throughout the duration of the task.

### Affective Go/NoGo task

**Fig. 1:**
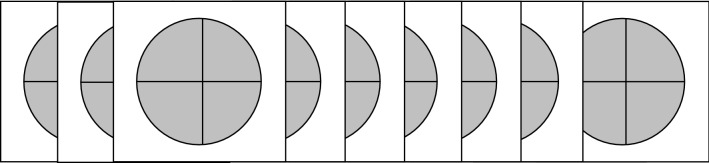
Learning phase: “standard” stimulus (700 ms) was presented 10 times in succession represented by a shape

Participants performed a temporal Go/NoGo task using emotional stimuli, adapted from two primary studies [27, 36]. It was comprised of a learning phase, a practice phase, and a testing phase. During the learning phase, participants were shown the “standard” stimulus duration (700 ms) 10 times, represented by a gray oval on the screen that was the same size as the actual stimuli (Fig. 1).

**Fig. 2:**
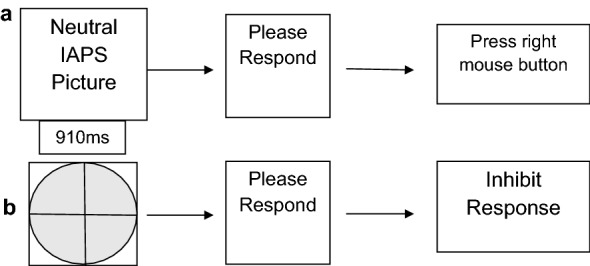
Practice phase. **a** If the participant is presented with the target stimulus (in the example above, the target stimulus is a neutral IAPS picture), the participant will judge if the stimulus is shorter or longer than the standard duration. In the example above, the participant should press the right button on the mouse to indicate that the duration was longer than the standard stimulus duration. **b** If the participant is presented with the nontarget stimulus (the gray oval used in the learning phase), the participant will inhibit any response and wait for the next stimulus presentation

During the practice phase, participants learned the Go/NoGo paradigm using neutral stimuli for both target and nontarget stimuli. The target stimuli were neutral IAPS pictures, while the nontarget stimulus was the gray oval used during the learning phase. In its entirety, the practice phase consisted of one trial block with 150 presentations of target stimuli (30 presentations of each duration condition) and 50 presentations of nontarget stimuli; however, participants were only exposed to 7 min of the practice phase in order to allow enough time for them to gain mastery of the task without becoming bored or lethargic. Stimuli were presented in five stimulus durations (280, 490, 700, 910, and 1120 ms). The occurrence of target and nontarget stimuli was pseudo-random, and the interstimulus interval was 1600 ms. The participants compared the duration of the target stimulus presentation to the “standard” duration. The participants then responded using a mouse according to the comparison made. If the participants made the judgment that the target stimulus duration was longer than the “standard” duration, the participants were instructed to press the right mouse button using the third finger of the right hand. If the target stimulus was perceived as being shorter than the “standard” duration, the participant was instructed to press the left mouse button using the index finger of the right hand. Even though some target stimuli were equal in duration to the “standard” stimulus duration, participants were forced to choose between only two responses (longer than or shorter than the “standard”). This allowed for testing the effect that personality traits and/or emotion had on time estimation (Fig. 2).

**Fig. 3:**
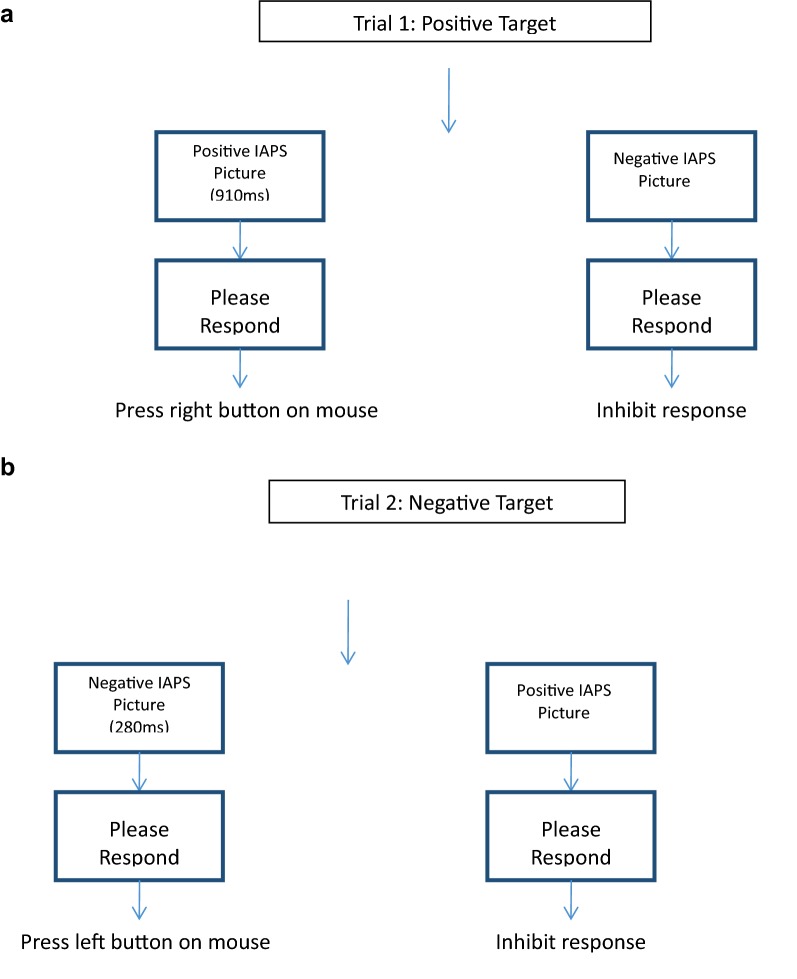
Test phase. **a** During the Positive Target Trial Block, if the participant is presented with a target stimulus (positive IAPS picture), the participant will compare its duration to the “standard” duration. The participant will then respond using the mouse as was learned during the practice phase. In the example above, the participant should judge the duration to be longer than the “standard,” and thus press the right button on the mouse. When presented with a negative (nontarget) stimulus, the participant should inhibit a response. **b** During the Negative Target Trial Block, if the participant is presented with a target stimulus (negative IAPS picture), the participant will compare its duration to the “standard” duration and then respond using the mouse. In the example above, the participant is presented with a “shorter” stimulus and thus should respond by pressing the left button on the mouse. When presented with a positive (nontarget) stimulus, the participant should inhibit a response

During the testing phase, participants encountered two trials of the previously described Go/NoGo task, in which target stimuli were based on valence (positive or negative). During one trial block, positive IAPS pictures served as target stimuli with negative IAPS pictures acting as the nontarget stimuli. During this trial block, participants chose if a positive stimulus was shorter than or longer than the “standard” duration, and inhibited any response to negative stimuli (Fig. 3a). During the other trial block, negative IAPS pictures were the target stimuli while positive IAPS pictures were nontarget stimuli. Participants chose if a negative stimulus was shorter than or longer than the “standard” duration during this trial block, and inhibited any response to positive stimuli presentation (Fig. 3b). The order of the positive and negative target sessions was counterbalanced across participants. The target stimuli were presented 150 times, while nontarget stimuli were presented 50 times. The occurrence of target and nontarget stimuli within each block was pseudo-random, and the interstimulus interval was 1600 ms. Each block contained 200 trials. The duration conditions were the same as those explained in the practice phase, and participants only had two possible response choices for target stimuli (longer than or shorter than the “standard”). Participants were encouraged to respond as quickly as possible to target stimuli through written and verbal instructions prior to task completion. Participants were presented with the “standard” duration five times between blocks.

### Procedures

Participants were tested in the Cognitive Neuroscience Laboratory located within the Department of Psychology at East Carolina University. Prior to participation, informed consent forms that were approved by the University Policy and Review Committee on Human Research of East Carolina University were reviewed orally with each participant and signed by each participant. Adherence to the “Ethical Principles of Psychologists and Code of Conduct” was kept with all participants in this study [37]. Once consent was established, participants completed self-report inventories and were acclimated to EEG recording procedures and given written instructions for the Affective Go/NoGo Task.

Procedures for electroencephalogram (EEG) analysis were adapted from Everhart and Demaree [38]. Participants were seated in an electrically shielded room in a comfortable reclining chair and fitted with a lycra electrode cap (Electro-Cap International, Inc.). Electrodes were arranged according to the 10–20 international system [39]. EEG data were recorded from 32 active electrode sites using linked ears (A1 and A2) as a reference (monopolar montage). Electrode placement included frontal: F3, F4, F7, F8; central: Cz, C3, C4; temporal: T3, T4, T5, T6; parietal: Pz, P3, P4; and occipital: O1, O2. In addition, electrodes were placed on the outer cantus of each eye so that eye movement recordings could be obtained. Electrode impedance was maintained below 5000 holms and checked at the beginning and end of the experimental session. Eye movement recordings were used to correct for the presence of eye movement artifact in the ERPs and to determine which trials should be excluded from averaging. Individual trials that contained excessive artifact associated with body and eye movement were excluded during off-line processing and prior to averaging. The EEG and eye movements were recorded with a bandpass of 1 and 100 Hz and a sensitivity of 7.5 µV/mm for EEG recordings. The EEG signal was amplified and converted on line to digital using a NeuroScan 32-channel PC-based EEG/evoked potential brain mapping system. A high-pass filter was used to eliminate slow wave frequencies that were less than 2 Hz. A 60 Hz notch filter was used to eliminate 60 Hz line noise. Artifact reduction was completed prior to computing grand averages for EEG and N2 data. The EEG data were converted on line for display, storage, and analysis [38].

Once participants finished reading the instructions for completing experimental procedures, baseline EEG was recorded according to procedures adapted from Davidson [40] including four minutes of baseline recording alternating between eyes open and eyes closed conditions. Participants then participated in the learning, practice, and test phases of the affective Go/NoGo task. Before each trial of the test phase, participants engaged in the learning phase. Error rate was measured as a behavioral variable to assess a bias in time perception during the “Go” standard duration stimuli presentations. After completion of all trials, the N2 responses were identified by visual inspection as the most negative peak between 100 and 300 ms [27]. Difference waves between Go and NoGo stimuli of equal duration for each valence were computed to form the N2d component (NoGo–Go). Separate grand averages for all data were created. Event-related potentials were averaged across participants for emotional valence and stimulus duration.

### Analyses

#### Hypothesis one

Correlation analyses were performed to determine the relationship between BIS, BAS, and an overestimation bias score when presented with target stimuli that were equivalent to the “standard” duration. The overestimation bias score was computed as the proportion of “longer” responses to the overall number of responses made during each test phase trial. The distribution of these scores was normal. These analyses were used to investigate the hypothesis that higher self-reported BIS scores would be associated with the tendency to overestimate the amount of time that negative stimuli were presented. These analyses were also used to investigate the hypothesis that higher self-reported BAS scores would be associated with the tendency to overestimate the amount of time that positive stimuli were presented.

#### Hypothesis two

Paired samples t tests were used to investigate the hypothesis that N2 amplitudes for “NoGo” stimuli would be larger than N2 amplitudes for “Go” stimuli. ANCOVA with BIS/BAS as covariates and the dependent variable of N2d amplitude (NoGo–Go N2 amplitude for emotion and duration condition) was also conducted. Duration (short and long) and valence (positive and negative) were included as factors. These analyses were used to investigate the hypothesis that higher BAS scores are associated with greater N2 amplitudes for positive NoGo stimuli. These analyses were also used to investigate the hypothesis that higher BIS scores are associated with greater N2 amplitudes for negative NoGo stimuli.

## Results

Statistical analyses were conducted using SPSS 19 statistical software package (IBM, Inc., Armonk, NY). Raw data were initially inspected for missing data and normality. Behavioral data from seven participants were incomplete due to noncompliance with the task and were left out of correlation analyses for hypothesis one. Due to substantial electrooculography (EOG) and electromyography (EMG) artifact during ERP recordings, nineteen participants were excluded from ANCOVA for hypothesis two. EOG and EMG were related to researchers’ observations of participants shifting in their seat and a considerable amount of yawning behaviors.

### Hypothesis one: relationships between BIS, BAS, and time perception

Results for evaluation of assumptions of normality indicated a positively skewed leptokurtic distribution of BAS Reward Responsiveness, which was corrected by excluding two univariate outliers on BAS Reward Responsiveness from analysis. This and initial exclusions due to noncompliance with the task resulted in 36 participants for correlation analysis.

To determine the relationship between BIS, BAS, and overestimation tendencies according to stimulus valence, directional correlation analyses were performed. Basic descriptive statistics and zero-order correlation coefficients between BIS, BAS subscales, and overestimation bias scores are presented in Table 1. Self-reported BAS Total (BAS TOT) scores (*M* = 21.91, SD = 5.13) were significantly, positively correlated with overestimation bias scores (OEPos) for positive stimuli (*M* = 49.35, SD = 24.70), *r* =.292, *n* = 36, *p* = .0421, 90% CI [.014, .53]. Self-reported BAS Drive (BAS D) scores (*M* = 10.07, SD = 3.22) were significantly, positively correlated with OEPos (*M* = 49.35, SD = 24.70), *r* =.312, *n* = 36, *p* = .0320, 90% CI [.036, .54]. These findings support the hypothesis that higher BAS scores would be associated with the tendency to overestimate positive “Go” stimuli. On the other hand, self-reported BIS scores (*M* = 15.42, SD = 3.73) were not significantly correlated with overestimation bias scores (OENeg) for negative stimuli (*M* = 53.068, SD = 27.49), *r* =.056, *n* = 36, *p* = .373, 95% CI [− .277, .377]. There was insufficient evidence to support the hypothesis that higher BIS scores would be associated with the tendency to overestimate negative “Go” stimuli.

**Table 1:** Correlation matrix showing relationships between BIS Total, BAS Total, BAS subscales, and overestimation bias scores

	OEPos	OENeg	BIS	BAS			
				TOT	RR	D	FS
BAS							
FS							
D							.464**
RR						.187	.325*
TOT					.440**	.874**	.811**
BIS				− .019	.171	− .131	.073
OENeg			.056	.212	.110	.262	.063
OEPos		.574**	.155	.292*	.025	.312*	.186
Mean	49.352	53.068	15.420	21.910	4.580	10.070	7.260
SD	24.696	27.487	3.730	5.131	.879	3.217	2.381

*BIS* behavioral inhibition system total, *BAS TOT* behavioral activation system total, *BAS RR* behavioral activation system reward responsiveness, *BAS D* behavioral activation system drive, *BAS FS* behavioral activation system fun seeking, *OEPos* overestimation bias scores positive Go stimuli, *OENeg* overestimation bias scores negative Go stimuli

*p < .05; **p < .01

To further investigate the relationship between BIS, BAS, and overestimation tendencies according to stimulus valence, correlation analyses were performed after stratifying data by sex. This was done in response to observations that women tended to have higher positive overestimation bias scores (*M* = 50.557, SD = 28.568) compared to men (*M* = 43.936, SD = 12.462), as well as higher negative overestimation bias scores (*M* = 54.783, SD = 28.677) compared to men (*M* = 47.943, SD = 23.813). There were also far fewer men than women who participated in this study, and most of the men participated over the summer as a way to earn extra credit in class, possibly making their motivation for participating in this study different than that of those who participated over the fall semester for course credit. Basic descriptive statistics and zero-order correlation coefficients between BIS, BAS subscales, and overestimation bias scores for women are presented in Table 2. Self-reported BAS D scores (*M* = 11.000, SD = 3.142) were significantly, positively correlated with OEPos (*M* = 50.557, SD = 28.568), *r* =.345, *n* = 28, *p* = .0360, 90% CI [.073, .57]. This finding supports the hypothesis that higher BAS scores would be associated with the tendency to overestimate positive “Go” stimuli. No other significant correlations were found. There was insufficient evidence to support the hypothesis that higher BIS scores would be associated with the tendency to overestimate negative “Go” stimuli.

**Table 2:** Correlation matrix showing relationships between BIS Total, BAS Total, BAS subscales, and overestimation bias scores for women

	OEPos	OENeg	BIS	BAS			
				TOT	RR	D	FS
BAS							
FS							
D							.583**
RR						.125	.446**
TOT					.605**	.808**	.877**
BIS				.473**	.494**	.346*	.281
OENeg			.206	.189	− .018	.275	.126
OEPos		.609**	.277	.258	− .077	.345*	.254
Mean	50.557	54.783	14.630	23.410	5.000	11.000	7.410
SD	28.568	28.677	4.030	6.026	2.140	3.142	2.500

*BIS* behavioral inhibition system total, *BAS TOT* behavioral activation system total, *BAS RR* behavioral activation system reward responsiveness, *BAS D* behavioral activation system drive, *BAS FS* behavioral activation system fun seeking, *OEPos* overestimation bias scores positive Go stimuli, *OENeg* overestimation bias scores negative Go stimuli

*p < .05; **p < .01

Table 3 presents correlation data between men’s self-reported BIS and BAS scores and overestimation bias scores. No significant correlations were found, indicating insufficient evidence to support hypothesis one.

**Table 3:** Correlation matrix showing relationships between BIS Total, BAS Total, BAS subscales, and overestimation bias scores for men

	OEPos	OENeg	BIS	BAS			
				TOT	RR	D	FS
BAS							
FS							
D							.431
RR						.543*	.468
TOT					.712**	.838**	.834**
BIS				− .285	− .103	− .562*	.038
OENeg			− .121	.269	.497	.299	.016
OEPos		.560*	− .031	.163	.203	.333	− .092
Mean	43.9356	47.943	18.310	20.230	4.770	8.000	7.460
SD	12.462	23.813	2.689	4.885	.927	2.483	2.570

*BIS* behavioral inhibition system total, *BAS TOT* behavioral activation system total, *BAS RR* behavioral activation system reward responsiveness, *BAS D* behavioral activation system drive, *BAS FS* behavioral activation system fun seeking, *OEPos* overestimation bias scores positive Go stimuli, *OENeg* overestimation bias scores negative Go stimuli

*p < .05; **p < .01

### Hypothesis two: personality, affective states, and the N2

To investigate the hypothesis that N2 amplitudes would be greater (more negative) in response to “NoGo” than to “Go” stimuli presentations, directional paired samples t tests were performed. Due to artifact, eight participants were excluded from this analysis, leaving *n* of 37. As expected, N2 amplitudes were significantly greater (more negative) in response to “NoGo” stimuli (*M* = − 7.136 microvolts, SD = 4.0364) than in response to “Go” stimuli (*M* = − 6.118 microvolts, SD = 3.379), *t*(36) = 1.886, *p* = 0.0335, 90% CI [.106, 1.929]. This finding supports the hypothesis that “NoGo” N2 amplitudes would be more negative than “Go” N2 amplitudes.

**Fig. 4:**
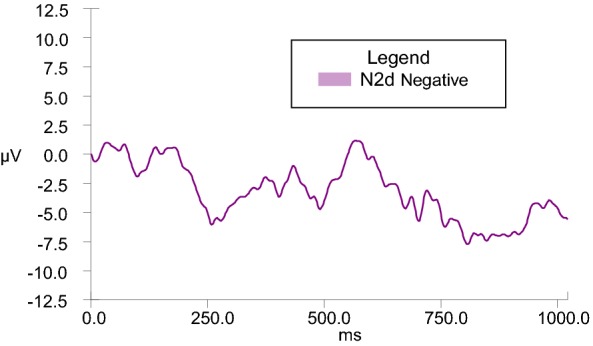
Representative N2d (NoGo–Go) grand average at scalp electrode Fz

N2d difference waves were calculated in order to serve as the dependent variable in analyses of covariance across Go and NoGo conditions. In order to enhance understanding, a graphic representative depiction of the N2d wave is observed in Fig. 4. While it is the N2d wave values that are used for analyses, the differences are appreciated in visual format via provision of separate grand averages of Go and NoGo data (as depicted in figures V–VIII). GLM ANCOVAs were conducted to evaluate the influence of emotional valence (positive or negative) and duration (280, 490, 700, 910, and 1120 ms) of stimuli presentation on N2 amplitude across Go and NoGo conditions while taking into consideration covariates of BIS and BAS personality traits. There was a significant emotional valence x BIS Total interaction, *F*(1, 20) = 7.028, *p* = .015 for 280-ms condition, and a significant emotional valence x BAS Total interaction, *F*(1, 22) = 4.602, *p* = .043 for 910-ms condition.

**Fig. 5:**
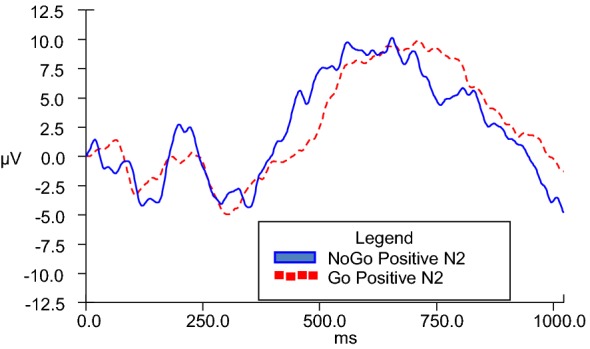
Go and NoGo N2 ERP grand averages for 280-ms positive condition at electrode FZ

**Fig. 6:**
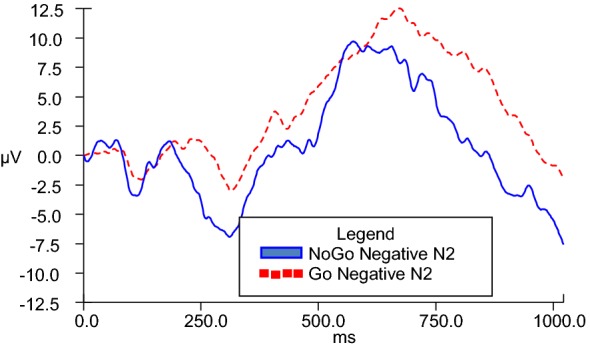
Go and NoGo N2 ERP grand averages for 280-ms negative condition at electrode FZ

No other main effects or interactions were observed. To examine the significant interactions observed for the 280-ms condition and the 910-ms condition, two separate post hoc correlation analyses were completed involving emotional valence (positive and negative) and corresponding scores on BIS and BAS. For the 280-ms condition, directional post hoc correlation analyses indicated that the N2d for positive stimuli at the 280-ms condition (P1611) (*M* = − 11.455 microvolts, SD = 16.648) had a strong zero-order correlation in the opposite direction as hypothesized with participants’ BIS Total self-report scores (*M* = 15.330 microvolts, SD = 3.397), *r* =.549, *n* = 24, *p* = .967, 95% CI [.187, .780], while the N2d for negative stimuli at the 280-ms condition (N1611) (*M* = − 10.962 microvolts, SD = 14.544) did not significantly or strongly correlate with BIS Total. Figure 5 illustrates NoGo and Go N2 amplitudes during the 280-ms duration condition for positive stimuli presentation, while Fig. 6 illustrates the same information for negative stimuli presentation.

**Fig. 7:**
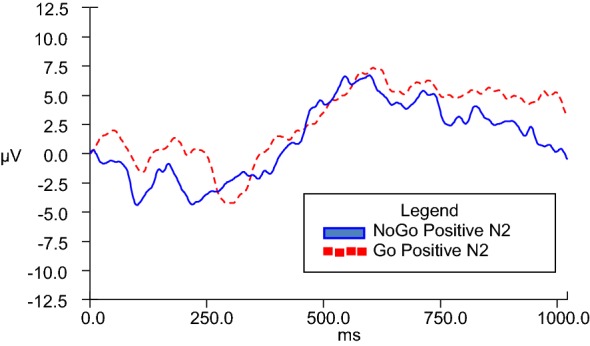
Go and NoGo N2 ERP grand averages for 910-ms positive condition at electrode FZ

**Fig. 8:**
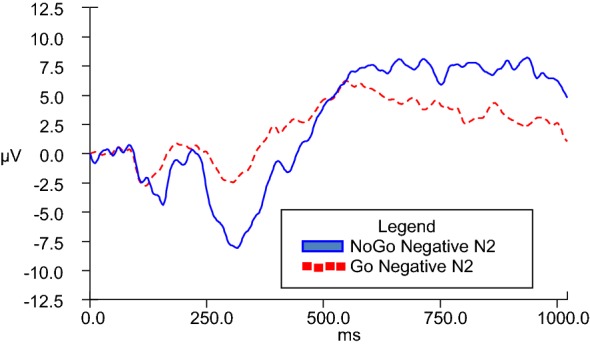
Go and NoGo N2 ERP grand averages for 910-ms negative condition at electrode FZ

For the 910-ms condition, directional post hoc correlation analyses indicated that the N2d for negative stimuli at the 910-ms condition (N1914) (*M* = − 10.846 microvolts, SD = 8.380) had a strong zero-order correlation in the opposite direction as hypothesized with participants’ BAS Total self-report scores (*M* = 23.230 microvolts, SD = 5.101), *r* =.496, *n* = 26, *p* = .995, 95% CI [.134, .741], while the N2d for positive stimuli at the 910-ms condition (*M* = − 11.591 microvolts, SD = 11.731) did not significantly or strongly correlate with BAS Total. These findings are in opposition to the hypothesis that greater BAS scores would be associated with increased N2d amplitudes for positive stimuli presentation. Figure 7 illustrates NoGo and Go N2 amplitudes during the 910-ms duration condition for positive stimuli presentation, while Fig. 8 illustrates the same information for negative stimuli presentation.

## Discussion

### Summary of results

The main findings related to hypothesis one included significant correlations between overestimation bias scores and BAS self-report scores. Hypothesis one posited that higher BAS scores would be associated with greater overestimation bias scores for positive stimuli presentation. (Based on previous findings in the literature that visual emotional stimuli evoke arousal, higher BAS scores are associated with sensitivity to reward and positive emotionality, and BIS is associated with sensitivity to anxiety, novelty, and punishment.) The second part of hypothesis one was that higher BIS scores would be associated with greater overestimation bias scores for negative stimuli presentation on the same premise. Higher BAS scores were associated with positive stimuli presentation. However, BIS scores were not significantly correlated with overestimation bias scores. BAS Drive subscale scores were main contributors to this partial support of hypothesis one, as the scores from BAS Drive were the only subscale scores that were significantly correlated with overestimation bias scores for positive stimuli. When data for hypothesis one were stratified by sex, women’s BAS Drive scores were significantly correlated with overestimation bias scores for positive stimuli presentation, while no such relationship was evidenced for men’s BAS subscale scores. This may indicate the need to test for sex-related differences in affective time perception according to personality traits in the future.

Support for the first part of hypothesis two was found, which stated that N2 amplitudes would be greater in response to “NoGo” than to “Go” stimuli presentations, indicating that the novel affective Go/NoGo task successfully elicited the N2 component thought to be associated with inhibition. Partial support for the second part of hypothesis two was observed. It was hypothesized that higher BIS scores would be associated with greater N2d difference waves for negative stimuli presentation and higher BAS scores would be associated with greater N2d difference waves for positive stimuli presentation. Indeed, N2d difference waves differentiated across personality trait levels; however, higher BIS Total scores were associated with higher N2d amplitudes during positive stimulus presentation for 280 ms, while higher BAS Total scores were associated with higher N2d amplitudes during negative stimuli presentation for 910 ms. These findings are different from previous findings indicating stronger neurophysiological responses of high BAS and BIS scorers to positive and negative stimuli presentation, respectively [22].

### Partial support for arousal-based models of time perception

Results from hypothesis one indicate the tendency to overestimate time duration was associated with higher BAS self-report scores, especially BAS Drive, during the presentation of positive stimuli. BAS Drive is associated with strong and quick persistence to obtain goals. Perhaps this trait in particular is a measure of baseline arousal levels on which people vary their perceptions of time passing for even very quick durations. It has been discussed in the literature that visual emotional stimuli evoke arousal, theoretically speeding up the internal clock via the pacemaker mechanism. Findings from the present study may suggest that BAS Drive trait is sensitive to the pacemaker. Making underestimations of time would have been supportive of attentional-based models of time perception, while making overestimations supported an arousal-based model of time perception [41–43].

From a clinical perspective, it is interesting to note that BAS is associated with overestimation of positive stimuli. Individuals with elevated BAS typically engage in positive, approach-related behavior and are generally thought of as less anxious or fearful than individuals with elevated BIS. Although only speculative, it is possible that individuals with elevated BAS are somewhat resilient to the effects of negative stimuli. In contrast, individuals with elevated BIS are thought to experience positive stimuli somewhat differently, to the extent that it could actually be perceived as negative. Although only in infant stages, there is a line of research that suggests that individuals with elevated BIS are less adherent to simple medical treatments (i.e., positive stimuli) that could improve quality of life and prevent long-term medical complications [44].

Greater N2 amplitudes for NoGo stimuli in general indicated an inhibitory response to emotionally incongruent stimuli as expected. The presence of the N2 indicates participants’ use of orbitofrontal and anterior cingulate cortices and reflects inhibition on a premotor level [45]. Since previous research indicated that higher BAS and BIS scores were associated with more intense orientation and responses to positive and negative stimuli, respectively, it was originally hypothesized that higher BAS self-report scores would be associated with greater N2d responses to positive stimuli, while higher BIS self-report scores would be associated with greater N2d responses to negative stimuli assuming an arousal-based model of time perception. However, BIS Total scores were associated with greater N2d responses to positive stimuli, perhaps suggesting that positive stimuli were being perceived as relatively novel experiences to participants’ general perception styles. BAS Total scores on the other hand were associated with greater N2d responses to negative stimuli, again suggesting an orientation to novel stimuli that were incongruent to participants’ general perception styles. These findings are contrary to arousal-based models of time perception and past research involving individual differences [46] and indeed may be indicative of attentional mechanisms involved in time perception.

Tipples [46] found support for arousal-based time perception models, in that negative emotionality was associated with overestimations of angry and fearful stimuli presentation durations. It was suggested that attentional effects were not observed in that study because they were mediated by emotional arousal through noradrenaline, which affects the operation of both attentional and time processes, and is also thought to facilitate orienting and slower disengagement of attention. Since the current study found results in opposition to arousal-based models of time perception, perhaps the Go/NoGo task tapped the previously described attentional mechanisms that were sensitive to both noradrenergic and dopaminergic pathways that are implicated in BIS and BAS, respectively. Of note, the Tipples [46] study differs fundamentally from the present study in two ways. First, the former study utilized affective faces rather than objects (i.e., IAPS). The negative affective faces were perceived as more arousing than positive affective faces. In the present study, the perceived levels of arousal for positive and negative stimuli were controlled. To this extent, the significant effects noted within Tipples’ [46] study may be attributable to the differences in magnitude of arousal between positive and negative affective faces. Second, Tipples [46] did not examine BIS and BAS; rather, the EAS Temperament Survey was used [47]. While this survey is associated with individual differences in positive and negative temperament and may overlap with BAS and BIS, there are inherent differences between these constructs that make direct comparison impossible.

Furthermore, findings indicated that higher BAS scores were associated with greater N2d amplitudes at the negative 910-ms duration condition (longer than the standard duration), while higher BIS scores were associated with greater N2d amplitudes at the positive 280-ms duration condition (shorter than the standard duration). Assuming that the Go/NoGo task was able to tap attentional mechanisms along with their respective neurophysiological pathways, perhaps individuals who report higher BAS are more sensitive to attentional mechanisms at relatively longer durations of incongruent emotional stimuli than higher BIS scorers.

### Limitations of current study

A major limitation to the present study was the inability to compare emotional conditions to neutral conditions. Including a neutral condition in future studies may help researchers isolate further arousal mechanisms associated with emotion. Another limitation was the amount of artifact encountered by taking N2d difference waves for hypothesis two. Increasing power in future studies by including more participants to account for this artifact may help detect findings the present study was unable to uncover. Previous research has included the use of a feedback tone for slow responses to “Go” stimuli, which helps to elicit the N2 ERP more reliably and effectively [28]. The last main limitation to this study was the sampling bias of including summer semester students who were also student athletes. Compared to women, a larger proportion of these student athletes were men, and stratifying data by sex for hypothesis one resulted in more consistent findings for women than men. This finding could also be the result of lower power for male participants in this sample. Regardless, sex-related differences in time perception should be explored in future studies.

### Conclusions

In summary, the hypotheses of this study were partially supported. BAS scores were associated with overestimation bias scores for positive stimuli. Higher BIS Total scores were associated with higher N2d amplitudes during positive stimulus presentation for 280 ms, while higher BAS Total scores were associated with higher N2d amplitudes during negative stimuli presentation for 910 ms. This study represents an initial attempt to understand the relationship between approach-avoidance tendencies and time perception via the utilization of a Go/NoGo ERP laboratory paradigm. Future studies will remedy the described limitations of the current investigation, with particular focus on examination of arousal mechanisms.

## References

[1] Burle B, Casini L (2001). Dissociation between activation and attention effects in time estimation: implications for internal clock models. J Exp Psychol Hum Percept Perform.

[2] Rueda AD, Schmitter-Edgecombe M (2009). Time estimation abilities in mild cognitive impairment and Alzheimer’s disease. Neuropsychology.

[3] Wearden JH (1999). “Beyond the fields we know…”: exploring and developing scalar timing theory. Behav Proc.

[4] O’Hanlon JF, McGrath JJ, McCauley ME (1974). Body temperature and temporal acuity. J Exp Psychol.

[5] Meck WH (1996). Dissecting the brain’s internal clock: how frontal-striatal circuitry keeps time and shifts attention. Brain Cogn.

[6] Treisman M, Faulkner A, Naish PL, Brogan D (1990). The internal clock: evidence for a temporal oscillator underlying time perception with some estimates of its characteristic frequency. Perception.

[7] Zakay D, Block RA (1995). Temporal cognition. Curr Dir Psychol Sci.

[8] Ohman A, Lundqvist D, Esteves F (2001). The face in the crowd revisited: a threat advantage with schematic stimuli. J Pers Soc Psychol.

[9] Russell JA, Mehrabian A (1977). Evidence for a three-factor theory of emotions. J Res Pers.

[10] Angrilli A, Cherubini P, Pavese A, Manfredini S (1997). The influence of affective factors on time perception. Percept Psychophys.

[11] Droit-Volet S, Brunot S, Niedenthal PM (2004). Perception of the duration of emotional events. Cogn Emot.

[12] Effron DA, Niedenthal PM, Gil S, Droit-Volet S (2006). Embodied temporal perception of emotion. Emotion.

[13] Gil S, Niedenthal PM, Droit-Volet S (2007). Anger and time perception in children. Emotion.

[14] Noulhiane M, Mella N, Samson S, Ragot R, Pouthas V (2007). How emotional auditory stimuli modulate time perception. Emotion.

[15] Carver CS, White TL (1994). Behavioral inhibition, behavioral activation, and affective responses to impending reward and punishment the BIS/BAS Scales. J Pers Soc Psychol.

[16] Harmon-Jones E, Harmon-Jones C (2010). On the relationship of trait PANAS positive activation and trait anger: evidence of a suppressor relationship. J Res Pers.

[17] Demaree HA, Robinson JL, Everhart DE, Youngstrom EA (2005). Behavioral inhibition system (BIS) strength and trait dominance are associated with affective response and perspective taking when viewing dyadic interactions. Int J Neurosci.

[18] Sutton SK, Davidson RJ (1997). Prefrontal brain asymmetry: a biological substrate of the behavioral approach and inhibition systems. Psychol Sci.

[19] Everhart DE, Carpenter MD, Carmona JE, Ethridge AJ, Demaree HA (2003). Adult sex-related P300 differences during the perception of emotional prosody and facial affect. Psychophysiology.

[20] Harmon-Jones E, Allen JJB (1998). Anger and frontal brain activity: EEG asymmetry consistent with approach motivation despite negative affective valence. J Pers Soc Psychol.

[21] Gray JA (1990). Brain systems that mediate both emotion and cognition. Cogn Emot.

[22] Balconi M, Falbo L, Brambilla E (2009). BIS/BAS responses to emotional cues: self report, autonomic measure and alpha band modulation. Personality Individ Differ.

[23] Coan JA, Allen JJ (2003). Frontal EEG asymmetry and the behavioral activation and inhibition systems. Psychophysiology.

[24] Demaree HA, Everhart DE, Youngstrom EA, Harrison DW (2005). Brain lateralization of emotional processing: historical roots and a future incorporating “dominance”. Behav Cogn Neurosci Rev.

[25] Coles MG, Rugg MD, Rugg MD, Coles MG (1995). Event-related brain potentials: an introduction. Electrophysiology of mind: event-related brain potentials and cognition.

[26] Beste C, Dziobek I, Hielscher H, Willemssen R, Falkenstien M (2009). Effects of stimulus-response compatibility on inhibitory processes in Parkinson’s disease. Eur J Neurosci.

[27] Falkenstein M, Hoormann J, Hohnsbein J (2002). Inhibition-related ERP components: variation with modality, age, and time-on-task. J Psychophysiol.

[28] Falkenstein M, Hoormann J, Hohnsbein J (1999). ERP components in Go/Nogo tasks and their relation to inhibition. Acta Physiol (Oxf).

[29] Burle B, Vidal F, Tandonnet C, Hasbroucq T (2004). Physiological evidence for response inhibition in choice reaction time tasks. Brain Cogn.

[30] Yu F, Yuan J, Luo Y (2009). Auditory-induced emotion modulates processes of response inhibition: an event-related potential study. NeuroReport.

[31] Coren S, Proac C, Duncan P (1979). A behaviorally validated self report inventory to assess four types of lateral preferences. J Clin Neuropsychol.

[32] Spinella M (2007). Normative data and a short form of the Barratt Impulsiveness Scale. Int J Neurosci.

[33] Donnellan MB, Oswald FL, Baird BM, Lucas RE (2006). The Mini-IPIP Scales: tiny-yet-effective measures of the big five factors of personality. Psychol Assess.

[34] Gilchrist H, Povey R, Dickinson A, Povey R (1995). The Sensation Seeking Scale: its use in a study of the characteristics of people choosing ‘adventure holidays’. Personality Individ Differ.

[35] Bradley MM, Lang PJ, Coan JA, Allen JJB (2007). The international affective picture system (IAPS) in the study of emotion and attention. Handbook of emotion elicitation and assessment.

[36] Gan T, Wang N, Zhang Z, Li H, Luo Y (2009). Emotional influences on time perception: evidence from event-related potentials. NeuroReport.

[37] American Psychological Association (2002). Ethical principles of psychologists and code of conduct. Am Psychol.

[38] Everhart DE, Demaree HA (2003). Healthy high hostiles evidence low alpha power (7.5–9.5 Hz) changes during negative affective learning. Brain Cogn.

[39] Jasper J (1958). Report of the committee on methods of clinical examination in electroencephalography. Electroencephalogr Clin Neurophysiol.

[40] Davidson RJ (1988). EEG measures of cerebral asymmetry: conceptual and methodological issues. Int J Neurosci.

[41] Davidson WB, House WJ (1982). Personality and the perception of time: a multimethod examination. Psychology.

[42] Rammsayer TH (1997). On the relationship between personality and time estimation. Personality Individ Differ.

[43] Zakay D, Lomranz J, Kaziniz M (1984). Extraversion-introversion and time perception. Personality Individ Differ.

[44] Moran AM, Everhart DE, Wuensch KL, Davis CE, Lee DO, Demaree HA (2011). Personality correlates of adherence with continuous positive airway pressure (CPAP). Sleep Breath.

[45] Bekker EM, Kenemans JL, Verbaten MN (2005). Source analysis of the N2 in a cued Go/NoGo task. Cogn Brain Res.

[46] Tipples J (2008). Negative emotionality influences the effects of emotion on time perception. Emotion.

[47] Buss AH, Plomin R (1984). Temperament: early developing personality traits.

